# Ambient Concentrations of Metabolic Disrupting Chemicals and Children’s Academic Achievement in El Paso, Texas

**DOI:** 10.3390/ijerph13090874

**Published:** 2016-09-01

**Authors:** Stephanie E. Clark-Reyna, Sara E. Grineski, Timothy W. Collins

**Affiliations:** 1 Department of Sociology and Anthropology, Northeastern University, 360 Huntington Avenue, Boston, MA 02115, USA; clark.stepha@husky.neu.edu; 2 Department of Sociology and Anthropology, University of Texas at El Paso, 500 West University Avenue, El Paso, TX 79902, USA; twcollins@utep.edu

**Keywords:** environmental justice, body mass index, obesity, obesogen, children, academic achievement, metabolic disruptors, endocrine disrupting chemicals, NATA

## Abstract

Concerns about children’s weight have steadily risen alongside the manufacture and use of myriad chemicals in the US. One class of chemicals, known as metabolic disruptors, interfere with human endocrine and metabolic functioning and are of specific concern to children’s health and development. This article examines the effect of residential concentrations of metabolic disrupting chemicals on children’s school performance for the first time. Census tract-level ambient concentrations for known metabolic disruptors come from the US Environmental Protection Agency’s National Air Toxics Assessment. Other measures were drawn from a survey of primary caretakers of 4th and 5th grade children in El Paso Independent School District (El Paso, TX, USA). A mediation model is employed to examine two hypothetical pathways through which the ambient level of metabolic disruptors at a child’s home might affect grade point average. Results indicate that concentrations of metabolic disruptors are statistically significantly associated with lower grade point averages directly and indirectly through body mass index. Findings from this study have practical implications for environmental justice research and chemical policy reform in the US.

## 1. Introduction

In recent years, environmental justice (EJ) researchers have examined the relationship between exposure to air toxics and children’s school performance at the level of the school [1,2,3,4,5,6,7]. These studies have found a negative association between school-based exposure to air toxics and academic performance, usually measured as aggregated standardized test scores and/or rate of absenteeism. One of the first studies used United States Toxic Release Inventory (TRI) data and 1990 census tract-level estimates of respiratory air toxics risk to predict standardized test scores in Los Angeles Unified School District (LAUSD) schools [6]. They found that air pollution levels at schools negatively and statistically significantly predicted standardized test scores, adjusting for school demographics. In a follow up study that included all public schools in California, Pastor et al. [7] found that the general pattern observed in the LAUSD held for the rest of the state. Outside of California, similar findings have been found in Baton Rouge, Louisiana [3,4] and Massachusetts [8]. Using the USEPA’s (United States Environmental Protection Agency’s) 2002 Risk-Screening Environmental Indicators (RSEI) geographic microdata, Lucier et al. [3] identified twelve known developmental, neurological and respiratory toxicants and found that the levels of these pollutants at school were linked to decreased test scores. Considerable research shows that respiratory and neurological toxins have negative impacts on school performance [3,4,5,6,7], as does diesel particulate matter (PM) [9,10]. To date, no research has examined the influence of ambient concentrations of metabolic disrupting chemicals on academic achievement.

Metabolic disrupting chemicals, or metabolic disruptors (MDs) as they are henceforth called, are important to consider because of the widespread use of these chemicals and the insufficient knowledge about the health effects of long term, low dose exposures. MDs are a specific class of endocrine disrupting chemicals (EDCs) that remain understudied despite growing evidence linking them to serious negative health consequences. Common MDs are bisphenol A, diethylstilbestrol (DES), phthalates, and organotins. MDs were first known as “obesogens” when scientists discovered that some EDCs predisposed laboratory mice to gain weight. In the last ten years, the term “metabolic disruptors” has become more common than “obesogens” because the effects of these chemicals extend beyond only weight gain and include altered liver and cardiovascular function and altered glucose homeostasis [11].

As endocrine disrupting chemicals, MDs interfere with the endocrine hormonal system in human and wildlife species and alter bodily functions [12]. They have been linked to obesity and also have negative impacts on neurological and thyroid function, insulin, metabolism, and glucose function/regulation, as well as adverse reproductive outcomes such as cancer of reproductive organs, endometriosis, infertility, and birth defects [12,13]. MDs, like other ECDs, function to mimic naturally occurring hormones such as estrogens, androgens, and thyroid hormones.

The link between environmental chemicals and weight gain is relatively novel [14], and evidence confirming the link between exposure to MDs and obesity has mounted in the past two decades [15,16,17]. Understanding the implications of exposure to MDs at the individual and population level is critical, because the use of MDs is widespread and highly unregulated; MDs are in food, pharmaceuticals, household products, and air emissions from a variety of sources. Many of these chemicals bioaccumulate and persist in the environment and have been detected in most people who have participated in biomonitoring studies [18]. We hypothesize that there may be a link between ambient outdoor air concentrations of MDs and individual children’s academic achievement. Previous studies linking air pollution to academic achievement have focused on explaining the association through respiratory health effects e.g., missing school due to asthma attacks [6,7] or through neurological effects on children’s development [2,3,4,10,19]. We propose an additional possible explanation, which is that MDs influence children’s weight, which negatively impacts their school performance. This is informed by studies finding a negative association between higher body mass indexes and reduced school performance in children [20,21,22,23].

The evidence for this link between children’s weight and school performance is not as well documented as the non-cognitive effects of obesity (e.g., glucose intolerance, high blood pressure, high cholesterol, sleep apnea, depression, and anxiety) [24,25]. Findings connecting obesity in childhood to academic outcomes are beginning to appear in the literature and some studies assert that the linkage is neurological as opposed to social/behavioral [26,27,28,29]. Recently, two studies found that childhood obesity was associated with poorer working memory performance and a decreased ability to regulate one’s cognitive control network, which includes inhibition, working memory, and cognitive flexibility [29,30]. This is significant because the relationship between the cognitive control network and school performance is well-documented [31]. While a neurological link is probable, there are also social and behavioral factors that likely influence the relationship between weight and school performance, such as bullying, discrimination from peers and teachers, and low self-esteem [32,33,34,35,36].

This study on MDs and children’s school performance builds from a large body of environmental health and environmental justice (EJ) scholarship. In the US, the academic EJ literature has shown that environmental risks have disproportionately fallen on minorities and the poor, and that race and class are often the greatest predictors of exposure to hazardous air pollutants in the US [37,38]. Previous school-based EJ studies have not examined MDs as an environmental hazard nor have they included body weight as a mediating variable in the relationship between residential exposure to air toxics and academic achievement. Specifically, we examine the impacts of ambient MDs at children’s home sites on grade point averages among a representative sample of 1319 fourth and fifth graders in El Paso, Texas directly and indirectly through body mass index using a mediation model.

### Research Questions and Proposed Model

The research questions and hypotheses are: (1) What is the direct effect of residential MD concentration on children’s grade point average (GPA), controlling for relevant covariates (e.g., economic deprivation, mother’s education, mother’s English proficiency, and teenage motherhood)? We hypothesize that MD concentration will be negatively associated with children’s GPA; (2) What is the direct effect of body mass index (BMI) on children’s GPA, controlling for relevant covariates? We hypothesize that BMI will be negatively associated with children’s GPA; (3) Does BMI mediate the association between residential MD concentration and children’s GPA, controlling for relevant covariates? We hypothesize that while MDs will have a direct effect on GPA, this effect will be mediated by BMI.

## 2. Materials and Methods

### 2.1. Study Context

The study took place in El Paso, Texas, USA. El Paso is located on the US-Mexico border, and has a population of 833,487 [39]. The population is 81% Hispanic (compared to 38% in Texas and 17% in the US). About 14% of El Paso residents are non-Hispanic white, while 4% are non-Hispanic black. El Paso has a rate of poverty (23% in 2011, when the survey was conducted) that is considerably higher than the national average (17%). Among El Paso residents, 26% are foreign-born, 15% are not US citizens, and 73% of the population over the age of five speaks a language other than English (predominately Spanish) at home.

High body mass indexes (BMIs) and being overweight are health concerns in El Paso County [40,41]. El Paso County has a lower percentage of obese adults (24%) than Texas (29%) and the US (27%). However, the county has a higher percentage of overweight adults (39%) compared to Texas (37%) and the US (36%) [40].

El Paso is also a city that struggles with air pollution problems, and this includes ambient MDs. The use of MDs is extensive and many of these chemicals are emitted into the air through polluting facilities and among other sources of air pollution. Diesel exhaust has been identified as a MD [42,43] and this pollutant is of great concern in El Paso. El Paso is home to a large binational trucking industry which experienced significant growth after the passage of the 1994 North American Free Trade Agreement. In 2014, nearly 760,000 trucks crossed from Ciudad Juárez (Mexico) through El Paso’s Ysleta-Zaragoza and Bridge of the Americas Ports of Entry [44], transporting items produced in Ciudad Juárez’s *maquiladora* industry. Two recent studies found that diesel PM was statistically significantly associated with decreased school performance in El Paso schoolchildren [9,10]. Apart from the trucking industry, El Paso is also home to numerous large scale polluting facilities such as Western Refining, Phelps Dodge Copper Products, and those associated with Fort Bliss and its US Army Air Defense Artillery Center, as well as a high flight volume international airport, all of which elevate levels of air pollution in El Paso County [45].

### 2.2. Data Collection

Data on socio-demographics, BMI, and academic achievement were collected through a cross-sectional mail survey that was sent to all caretakers of fourth and fifth graders in the El Paso Independent School District in 2012 [46]. The El Paso Independent School District (EPISD) is the tenth largest district in Texas and in 2012, there were over 64,000 students (K-12) enrolled in 94 campuses.

We used the Tailored Design Method to obtain the highest response rate possible [47]. We first sent out a survey package containing a consent form, English and Spanish versions of the survey, a return envelope, and a two-dollar incentive. The following week we sent out a bilingual reminder postcard to non-respondents, and the third week, we resent the package to all non-respondents. In total, 6295 surveys were delivered to the caretakers and we received 1904 responses, which gave us a response rate of 30 percent. Research has shown that comparable response rates can yield representative samples [48,49,50,51]. The sample was generally representative of EPISD fourth and fifth graders in terms of Hispanic ethnicity (82.2% versus 82.6%). The sample was slightly less economically poor as the percent of students qualifying for free or reduced price meals was 60.0% versus 71.3% among all EPISD fourth and fifth graders.

Information was gathered through the survey on both the primary and secondary caretakers of the child. Primary caretakers were 83% mothers and 10% fathers. Secondary caretakers were 57% fathers and 13% mothers. We drew from questions asked about the primary and secondary caretaker to create variables applicable to the child’s mother for the analysis, because mother’s attributes were the most commonly included covariates in a review of the literature on children’s academic achievement outcomes.

Six children were excluded from this analysis because they lived outside of the county limits and three children were excluded because of unconventional home and alternative schooling arrangements. 567 children were excluded from the sample due to substantial missing data for the analysis variables, leaving a total of 1319 children for analysis.

### 2.3. Variables

Descriptive statistics for all variables are presented in Table 1.

#### 2.3.1. Children’s Academic Performance

GPA is the dependent variable in this study and it was calculated using caretaker-reported grades from five different subjects (reading, language arts, math, social studies, and science) from the survey. The list of subjects and response options are identical to the official EPISD report cards and the subject grades were recoded so that F = 0; D = 1; C = 2; B = 3; and A = 4. The subject area scores were summed and divided by five to create the continuous GPA dependent variable. Children had a mean GPA of 3.3 out of 4.0 which is reflective of the national pattern for grades received in elementary school. According to the US Department of Education [52], 82.2% of students received either mostly A’s or mostly B’s nationwide in 2007. While not an exact match to these Department of Education figures, 78.4% of children in our sample had GPAs above 3.0.

#### 2.3.2. Concentration of Metabolic Disruptors

We used the USEPA’s 2005 National Scale Air Toxics Survey (NATA) census tract-level database to create the child-level MD values used in the analysis. The NATA includes all air toxics regulated by the US Clean Air Act (except criteria pollutants) that are known or suspected to cause cancer or neurological, respiratory and immunological diseases, as well as reproductive ailments [53]. NATA is currently the best available secondary data source for spatially explicit characterization of air toxics exposure risk in US metropolitan areas [54,55,56,57,58]. The USEPA works with states and industries to gather data about air toxics emissions and then compiles them in the NATA. Due to space restrictions, details pertaining to the NATA modeling procedures are provided elsewhere [59].

To generate the estimates for the ambient concentrations of MDs at each child’s home residence, we identified both known and suspected MDs from published lists and through a thorough review of toxicological and environmental health literature. Currently, the most comprehensive compilations of EDCs (including MDs) are provided by The Endocrine Disruption Exchange [60] and the Institute for Environment and Health [61], but these lists are not currently monitored or updated by agencies such as the USEPA. These lists include chemicals that are also included in the NATA [62]. A total of eight “known MDs” were identified in the 2005 NATA. These are arsenic, benzene, cadmium chloride, chlordane, dibutyl phthalate, diesel exhaust, ethylene glycol, and naphthalene. In the Endocrine Disruption Exchange [60], chemicals are grouped by various characteristics. Chemicals classified as “byproducts, intermediates, and reactants” are those that are used in the synthesis or creation of other chemicals and/or are the byproducts of these processes and are contaminants or impurities. “Solvents” are chemicals that are used to breakdown other chemicals. “Industrial additives” are used as surfactants, preservatives or antioxidants in industrial production processes [60]. As for the chemicals in our known MD variable, arsenic is an industrial additive and a byproduct/intermediate/reactant; cadmium chloride and chlordane are also industrial additives; diesel exhaust is a byproduct/intermediate/reactant; ethylene glycol and dibutyl phthalate are byproducts/intermediates/reactants, industrial additives, and solvents; benzene is a byproduct/intermediate/reactant and a solvent; lastly, naphthalene is a byproduct/intermediate/reactant [60]. While MDs are ubiquitous in the environment, due to data limitations, we are only able to examine MDs in air emissions that are captured by NATA. Another eight MDs were identified as “suspected MDs” and these are carbaryl, epichlorohydrin, ethylbenzene, lead compounds, styrene, phenol, nickel compounds, and selenium compounds.

**Figure 1:**
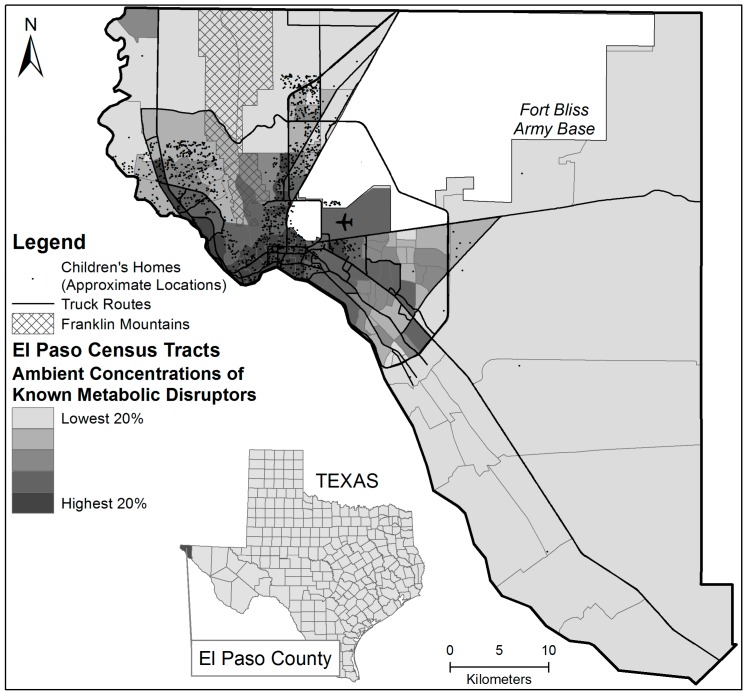
Levels of metabolic descriptor (MD) concentrations and approximate locations of participating children’s homes in the El Paso, TX Study Area.

For each MD, we used the “ambient concentration” measurement provided by the NATA to assess the level of the MD in each census tract. Ambient concentrations refer to raw concentrations of toxics in outdoor air. These concentrations are surrogate for exposures as they do not take into account human activity patterns (among other factors) as do the “risk” variables available in the NATA. These concentrations are additive so we summed the eight ambient concentrations to create a tract-level “known MD” variable, and then summed the 16 values together to create the “known or suspected MD” value. Next, using a Geographic Information System, we assigned each child the total ambient concentration of known and known or suspected MDs of the census tract in which he/she resided (based on home address). We report results using the “known MD” variable, and considered the “known or suspected MD” variable in a sensitivity analysis. The average ambient concentration of known MDs was 0.84 micrograms/m^2^ while the average ambient concentration of known and suspected MDs was 0.94 micrograms/m^2^. We used the natural log of the two MD variables to reduce skewness and kurtosis in the statistical analysis. Figure 1 depicts the distribution of known MDs in El Paso census tracts.

#### 2.3.3. Body Mass Index

We calculated BMI using caretaker reported height and weight values for the child. The weight in kilograms was divided by the square of the height in meters. It is the mediating variable in our model. In our sample, children had an average BMI of 19.59.

#### 2.3.4. Control Variables

In this study, we controlled for eight individual-level variables associated with children’s academic performance. Previous research has found that economic disadvantage is associated with decreased academic performance [63]. It is operationalized as (1) qualifying for free or reduced price meals (FRPM) at school [64]. We used this variable instead of poverty because it is a less conservative measure of socioeconomic disadvantage than is poverty. FRPM is 185% of the poverty line.

We controlled for (2) mother’s education (measured as years of schooling completed) because children of mothers with higher levels of education tend to perform better in school, compared to those of mothers with lower levels of education [65]. We controlled for (3) teenage motherhood because children born to younger mothers tend to fare worse in school as it is more challenging for these mothers to provide intellectually stimulating homes [65]. Our continuous mother’s age at the birth of her child variable was dichotomized into 1 = teenage mother (19 and younger) and 0 = not a teenage mother (20 years and older).

Having a (4) black/African American and/or (5) Hispanic mother has been linked with lower levels of academic performance among children. The academic achievement gap between students of color and white students has been well studied and documented [63,66,67,68] and is due to multiple factors including school tracking, institutional racism and discrimination, and parenting styles [66,67,69]. To determine whether the mother was Hispanic or non-Hispanic black, we drew from two questions that asked, “Are you of Hispanic, Latino, or Spanish origin?” and “What is your race?”

We controlled for (6) mother’s English proficiency because mothers who are not proficient in English may be unable to help with schoolwork [63]. Mother’s English proficiency was treated as a continuous indicator and was measured on a four-point scale (0 = not at all; 1 = not well; 2 = well; and 3 = very well). We also controlled for (7) children’s current age in years and the (8) sex of the child (0 = female; 1 = male).

In terms of our sample, the average BMI for children in our study was 19.59. Sixty percent of participating students qualified for free or reduced priced meals; 9% of children had a teenage mother at the time of their birth. Mothers had 13 years of education on average. Eighty percent of mothers were Hispanic while another 2% were non-Hispanic black. Lastly, mothers had an average score of 2.17 for English proficiency on a four-point scale. Because the sample only included fourth and fifth graders, the average age of the child was ten. Fifty percent of children were male while the other half were female.

### 2.4. Analysis Methods

To address non-response bias, we used multiple imputation techniques in IBM SPSS version 23 (IBM, Armonk, NY, USA). Multiple imputation (MI) is currently the best method to address missing data in quantitative analysis and is used to avoid bias that may occur when values are not missing completely at random [70]. We imputed missing values for ten data sets to increase power using a regression-based approach, and we specified 200 between-imputation iterations to ensure independence between the data sets [71]. Analyzing a single imputed data set would effectively treat the filled-in values as real data, so even the best imputation technique, when used with just one imputed data set, may underestimate sampling error. MI techniques appropriately adjust the standard errors for missing data [71]. We included all relevant variables in the multiple imputation procedure. The percent missing for the variables ranged from 0% to 12.7% (see Table 1). We pooled the data from each of the ten models in order to generate the results of our mediation analysis. We analyzed the originally ordinal measure (i.e., mother’s English proficiency) as a continuous predictor in the statistical model. This approach is considered a best practice in MI when imputing missing data and estimating model parameters, since rounding off imputed values based on discrete categorical specifications has been shown to produce more biased parameter estimates in analysis models [71].

For this paper, we ran bivariate correlations and then used the “PROCESS” macro [72] in SPSS to run a mediation model to examine if MDs operate through BMI (our mediating variable) to affect GPA. Mediation models are composed of two antecedent variables (*X* and *M*) and two consequent variables (*M* and *Y*). The *X* variable causally influences *Y* and *M*; *M* causally impacts *Y* [72]. In other words, mediation analyses are used to examine how one variable affects another variable through two pathways: through a direct pathway (*X* on *Y*) and through a mediating variable (*X* through *M* on *Y*) [72]. The mediating variable carries the influence of the independent variable on the dependent variable and the indirect effect is a measure of how much of the effect of the *X* variable on *Y* is mediated by *M*. We used the normal theory approach (i.e., the Sobel test) to test for the significance of the indirect effect of *X* on *Y*. The Sobel test is a specialized *t*-test that determines whether the change in the effect of the independent variable, after the inclusion of the mediator, is a statistically significant change and whether the mediator’s effect in the model is significant [72]. Previous research has found that exposure to environmental toxics negatively impacts school performance in children (e.g., [4,9]), but it is not yet known through which mechanism this association works. The mediation model allows us to examine one potential pathway, i.e., through BMI.

## 3. Results

### 3.1. Correlations

Correlations are presented in Table 2. BMI (*r* = −0.141, *p* < 0.01) and the outdoor air concentration of known MDs (*r* = −0.226, *p* < 0.01) were negatively associated with children’s GPA, as was the known and suspected MDs (*r* = −0.228, *p* < 0.01) variable. In terms of GPA and the control variables, qualifying for free or reduced priced meals (*r* = −0.316, *p* < 0.01) exhibited the strongest correlation with children’s GPA, followed by mother’s education (*r* = 0.298, *p* < 0.01). Mother being Hispanic (*r* = −0.201), having a teen mother (*r* = −0.100), being younger (*r* = −0.074), and being male (*r* = −0.096) were also associated (*p* < 0.01) with lower GPA.

Greater BMI was positively and significantly associated with known MD concentrations (*r* = 0.104, *p* < 0.01). Qualifying for free or reduced priced meals (*r* = 0.149, *p* < 0.01) exhibited the strongest correlation with BMI, followed by mother’s education (*r* = −0.106, *p* < 0.01). Having a Hispanic mother (*r* = 0.123, *p* < 0.01) was also positively associated with BMI.

In terms of the ambient outdoor air concentrations of known MDs, qualifying for free or reduced priced meals (*r* = 0.329, *p* < 0.01) exhibited the strongest correlation. Mother being Hispanic (*r* = 0.256, *p* < 0.01), Mother being non-Hispanic black (*r* = −0.068, *p* < 0.05), having a teen mother (*r* = 0.083, *p* < 0.01), and mother’s English proficiency (*r* = 0.136, *p* < 0.01) were also associated with concentrations of known MDs.

### 3.2. Mediation Model

**Figure 2:**
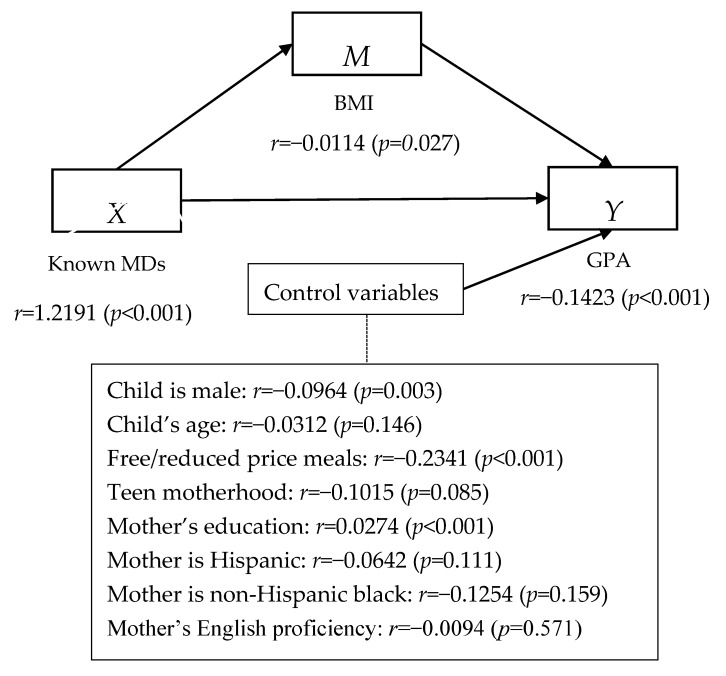
Pooled Results for PROCESS Model Predicting Children’s Grade Point Average (GPA) (*n* = 1319).

Results are shown in Figure 2. The effect of known MDs (*X*) on BMI (*M*) were positive and statistically significant (*r* = 1.2191, *p* < 0.01). The direct effect of known MDs (*X*) on GPA (*Y*) was negative and statistically significant (*r* = −0.0114, *p* = 0.03) and this effect was mediated by the indirect effect of BMI (*M*) on GPA (*Y*), which was negative and statistically significant (*r* = −0.1423, *p* < 0.01). Because the relationship between *X* and *M* is significant and positive, higher levels of known MDs are associated with higher BMI. The direct effect of *X* on *Y* is significant and negative meaning that higher levels of known MDs are associated with lower GPA. Lastly, the indirect effects of *X* on *Y* (through *M*) are statistically significant and negative, meaning that higher levels of known MDs negatively impact GPA through higher BMI. When using the known/suspected MD variable, instead of the known MD variable, results were identical in terms of direction and significance. Note those effects are significant adjusting for the full suite of control variables. In terms of the control variables, mother’s education was positively associated with GPA (*r* = 0.0274, *p* < 0.01), while being male (*r* = −0.0965, *p* = 0.03) and qualifying for free or reduced priced meals (*r* = −0.2341, *p* < 0.01) were associated with lower GPAs.

## 4. Discussion

This study is novel in that it is the first to address a limitation in the literature on pollution and academic performance, and the EJ literature more generally, with its focus on MDs. It is a first foray into examining the potential impacts of ambient concentrations of MDs on school performance. The relationship between exposure to hazardous air pollutants and academic achievement has been clearly documented [1,3,4,9,10], yet the pathways through which this relationship works remain unclear. Researchers have suggested that the relationship between exposure to air pollutants and decreased school performance can be explained in two ways [9,10]. First, exposure to air pollutants can be linked to respiratory illnesses and more missed school due to these illnesses. Second, the relationship can be explained through neurological impacts. Exposure to air toxicants may damage critical areas of children’s brains [73], resulting in reduced capacities to learn and retain information. In the study, we hypothesized that air toxics, specifically MDs, also have negative implications for children’s academic achievement both directly and indirectly through children’s BMI. This hypothesis encompasses three relationships, which we will discuss in turn.

The first relationship is the path connecting the concentrations of MDs at the child’s home to BMI, which was positive and statistically significant, supporting our hypothesis. This finding seems to substantiate the previously hypothesized environmental link between toxics and weight [11,14,15,16,17]. This finding is not surprising given that MDs disrupt the body’s endocrine and metabolic functioning, meaning that the children are more susceptible to weight gain, diabetes, and metabolic syndrome [11].

The second relationship is the path from BMI to GPA, which was negative and statistically significant, indicating that higher BMI was associated with lower GPA. In terms of why this association was found, there are currently two hypotheses in the literature. The first is a psychosocial explanation, which asserts that heavier children tend to face bullying and discrimination and have lower self-esteem, which translates into generally worse school outcomes [32,33,34,35,36]. The second is a biological explanation, which posits that obesity affects brain and memory functions, contributing to poor academic performance outcomes [26,27,28,29].

The third relationship is the direct effect of MD concentrations on GPA, which was negative and statistically significant. This reveals that although BMI significantly mediates the relationship between MDs and GPA, there is an additional direct effect whereby MDs predict lower GPAs, apart from their associations with BMI. This is likely because MDs harm bodies in myriad ways beyond promoting weight gain. The impacts of MDs even extend beyond metabolic and endocrine disruption to include, for example, neurological and developmental effects [11]; it is probable that these chemicals inflict additional damage that may degrade school performance.

These findings are important at both the individual and the population level. At the individual level, they are significant because school achievement predicts both future success in the labor market and health outcomes in adulthood [74]. The environmental justice literature has documented that poor and minority children are exposed to greater levels of air pollution than their white and more affluent counterparts [5,6,7,10,75]. Thus, it stands to reason that the greatest impacts of this insidious association between toxics and school performance are being borne by those who also struggle with other educational challenges. Additionally, it is unlikely that parents or teachers would notice small decreases in GPA and attribute them to either pollutants or BMI, making these findings particularly concerning and difficult to address through behavioral interventions. It suggests that few will take action to prevent exposure to these chemicals, since these associations will almost always go unrecognized.

At the population level, the bodily impacts of chemicals, including MDs, can have catastrophic effects. In addition to the growing cost of obesity and obesity-related diseases within the healthcare sector, Colborn [76] posited that decreased cognitive abilities due to chemical exposure could place a greater strain on remedial education while leading to the development of fewer people deemed intellectually “gifted.” Ultimately, population-level neurological and cognitive damage stemming from chemical exposures could place a dramatic burden on the healthcare sector and negatively impact the development of human capital, decreasing societal capacities for scientific and technological innovation.

### Limitations and Directions of Future Research

This study has several limitations. One relates to the measurement of MDs. There is not yet an official list of endocrine or metabolic disrupting chemicals, which impacts this study and others seeking to examine the effects of MDs on human and ecological well-being. Researchers with The Endocrine Disruption Exchange and the Institute for Environment and Health have provided lists of known and suspected MDs, but these are not monitored, regulated, or regularly updated by agencies such as the USEPA, US Centers for Diseases Control, or World Health Organization. Additionally, as of 2016, over 1000 EDCs have been identified [60]; the 2005 NATA includes data on only 177 chemicals which are all regulated by the Clean Air Act (Environmental Protection Agency 2014), many of which may have metabolic disrupting effects that are currently unknown. Because many chemicals in industrial use and on the consumer market are not regulated, many such chemicals now in circulation (or in development) may be identified as MDs for generations to come.

There are limitations with the USEPA’s NATA data. First, we paired our 2012 survey data with 2005 NATA estimates, which were the most recent estimates available when the study was conducted. Despite the time lag, we believe that the concentrations between 2005 and 2011 have remained relatively constant given that all major freeways, roads, factories, refineries, airports, train stations, and ports of entry within the EPISD have remained in the same locations since 2005. Secondly, this study examined only MDs that are emitted in the air and regulated under the Clean Air Act, and hence included in the NATA. Airborne emissions do not fully encapsulate the multiple forms of exposure to MDs, as MDs and EDCs are found in household and personal care products [77,78], in indoor air and dust [79], and food products and packaging [80,81], in addition to ambient air emissions.

Lastly, there are limitations associated with our survey data. We lack variables related to children’s health behaviors (e.g., exercise and diet), which would be important to control for in future studies. The effects of exposure to MDs, and EDCs more generally, are mediated by sleep patterns, the microbiome, preexisting health conditions, and other social and genetic determinants of health not included in our model [11]. The data used in our BMI calculations were reported by caretakers as opposed to collected through in-person measurements of the children. Research has shown that bias in height and weight reporting is greatest among parents of young children (ages 2–5) and reduced when they have older children [82]. Given that the children under study are ages 10–11, reporting bias is likely less of an issue than if we were studying preschoolers. Note also that researchers have concluded that parent-reported height and weight measures are accurate enough to be applied in studies on obesity in children ages 3–17 [83]. The use of cross-sectional survey data means that we examined associations at only one point in time and cannot determine if levels of MDs preceded decreased school performance.

## 5. Conclusions

The findings from this study report a disturbing association between probable chemical exposures to a new and relatively unregulated class of chemicals and academic performance in elementary school children. Children are more susceptible to the adverse effects of exposure to air toxics because they have larger lung surface in relation to their body weight compared to adults, meaning that they breathe in more air per kilogram of body weight. Additionally, children spend more time outside than adults do, and often engage in activities such as playing sports and running that require them to breathe in more air so their body weight burden is significantly greater than that of adults [84]. They also have more open pathways of environmental exposure (e.g., through hand-to-mouth contact) than do adults. These children, the majority of whom are ages 10–11, are in the pre-pubescent development stage. According to the developmental origins of disease theory, children at this stage of life are in a critical window whereby the body is much more sensitive to environmental exposures and stressors than it is at older ages; environmental exposures during these critical windows can radically alter gene development and, in turn, affect organ and tissue function [11,85]. Additionally, these environmental exposures are more likely to result in adverse health outcomes in children due to their rapidly developing systems. Exposure to MDs during this critical window of vulnerability may impact children’s abilities to learn and retain information.

These results contribute to discussions about the environment and obesity and the growing literature on children’s weight and academic achievement. Findings corroborate previous studies linking exposure to air toxics to academic achievement and demonstrate that, even after controlling for economic and demographic factors, MDs have negative impacts on children’s academic performance both directly and indirectly through BMI. The relevance of research on MDs is difficult to overstate, since rates of childhood obesity are expected to double in the next 20 years [26].

Research on MDs is in its infancy; in the future, more chemicals will be found to have metabolic disrupting properties, making such studies increasingly important. In terms of chemical-testing policy in the US, there is an urgent need to move beyond the cancer paradigm (i.e., a focus solely on cancer when testing a single chemical) and to develop innovative ways to test entire classes of chemicals (e.g., MDs) for multiple health effects at the same time. MDs have insidious effects on the endocrine system, which serves as the body’s “biological highway” [76]. Non-cancer outcomes including memory loss, decreased neurological capabilities, and behavioral disorders, such as autism and attention deficit/hyperactivity disorder, have serious consequences for human well-being. In order to reduce exposure to MDs and reduce the body burdens of toxic chemicals in humans, chemical manufactures could be required by US law to show “proof of safety” before chemicals are sold, as opposed to the current model, which allows chemicals to be sold until they are proven to cause adverse health effects. Researchers should also develop methods of monitoring both personal and community exposure to MDs across generations. The effects of environmental exposures may have a greater impact on health and non-health outcomes (including school performance) than genetic determinants, even though environmental determinants receive less focus and research funding.

## Figures and Tables

**Table 1:** Descriptive statistics for all analysis variables.

**Continuous Variables**	***N***	**% Missing**	**Min.**	**Max.**	**Mean**	**Standard Deviation**
Grade Point Average (GPA)	1240	6	0.20	4	3.40	0.70
Ambient concentration of known metabolic disrupters (MDs) (ln)	1319	0	−0.07	2.48	0.84	0.43
Known and suspected (ln) ambient concentration of MDs ^1^	1319	0	−0.02	2.61	0.94	0.44
Body Mass Index (BMI)	1273	3.50	6.67	46.48	19.59	5.16
Child’s age (years)	1312	0.50	9	13	10.40	0.80
Mother’s education (years)	1208	8.40	1	21	13.70	3.60
Mother speaks English	1180	12.70	0	3	2.20	1
**Dichotomous Indicators**	***N***	**% Missing**	**Frequency %**	**Yes**	**No**	
Child is male	1299	1.50	49.60	644	655	
Free/reduced priced meals	1209	8.30	40.90	494	715	
Teenage motherhood	1178	8.40	8.10	95	1083	
Mother is Hispanic	1185	10.20	76.70	909	276	
Mother is non-Hispanic black	1198	9.20	3.00	36	1162	

^1^ Used only in the sensitivity analysis.

**Table 2:** Correlation Matrix.

Variables	GPA	Known MDs	Suspected/Known MD	Child’s BMI	Child Is Male	Age of the Child	Free Meals	Teenage Motherhood	Mother’s Education	Mother Is Hispanic	Mother Is Non-Hispanic Black
GPA											
Known MDs (ln)	−0.226 **										
Suspected/known MDs (ln)	−0.228 **	0.999 **									
Child’s BMI	−0.141 **	0.104 **	0.103 **								
Child is male	−0.096 **	0.030	0.031	0.077 **							
Age of the child	−0.074 **	0.054 *	0.054 *	0.099 **	0.033						
Free meals	−0.316 **	0.329 **	0.328 **	0.149 **	−0.008	0.089 **					
Teenage motherhood	−0.100 **	0.083 **	0.088 **	−0.008	0.015	0.049	0.116 **				
Mother‘s education	0.298 **	−0.298 **	−0.299 **	−0.106 **	0.009	−0.075 **	−0.487 **	−0.079 **			
Mother is Hispanic	−0.201 **	0.256 **	0.257 **	0.123 **	−0.005	0.010	0.256 **	0.074 *	−0.238 **		
Mother is non-Hispanic black	0.016	−0.068 *	−0.067 *	−0.026	0.040	0.025	−0.067 *	0.020	0.060 *	−0.321 **	
Mother’s English proficiency	−0.066 *	0.136 **	0.137 **	0.042	0	0.048	0.112 **	0.012	−0.030	0.327 **	−0.078 **

** Correlation is significant at the 0.01 level (2-tailed). * Correlation is significant at the 0.05 level (2-tailed).
